# Antibodies to Brewer's Yeast in Rheumatoid Arthritis

**DOI:** 10.7759/cureus.4691

**Published:** 2019-05-17

**Authors:** Jack R Lichtenstein, Alan L Epstein

**Affiliations:** 1 Rheumatology, Anne Arundel Medical Center, Annapolis, USA; 2 Rheumatology, Pennsylvania Hospital, Philadelphia, USA

**Keywords:** rheumatoid arthritis, saccharomyces cerevisiae, mannan

## Abstract

Antibodies to brewer’s yeast or anti-Saccharomyces cerevisiae antibodies (ASCA) have been detected in 70% of patients with Crohn’s disease and have become a part of the evaluation of a patient for Crohn’s disease. Prior evaluation of these antibodies in rheumatoid arthritis have been inconsistent. In an initial small study, the levels of antibodies were elevated but not statistically significant. In a second large study from China, 40% of rheumatoid arthritis patients were positive for immunoglobulin A (IgA) ASCA and 20% positive for immunoglobulin G (IgG) ASCA.

Our study was inspired by the observation that several seronegative patients with rheumatoid arthritis were positive for ASCA antibodies. Between January 1, 2016 and January 1, 2018, a total of 241 patients with clinical rheumatoid arthritis were evaluated for antibodies to IGA and IGG ASCA, rheumatoid factor, cyclic citrullinated peptide (CCP), and antinuclear antibody (ANA). Our results indicate that 158 (66%) of these patients were positive for ASCA; 70 (29%) of these patients were positive for ASCA but negative for other serologies; 62% of the patients were positive for rheumatoid factor. Our results also indicate that the percentage of rheumatoid factor (95%) and CCP positive (78%) patients in the ASCA negative group was higher than the percentage of rheumatoid factor positive (49%) and CCP positive (37%) patients in the ASCA positive group, suggesting serologic differences between the two groups. Only 4% of the rheumatoid patients were negative for all the evaluated serologies.

The possible role of mannan, a mucopolysaccharide from the cell wall of Saccharomyces cerevisiae (S. cerevisiae) in producing rheumatoid arthritis is discussed.

## Introduction

Antibodies to brewer’s yeast or anti-Saccharomyces cerevisiae antibodies (ASCA) have been found to be present in 70% of patients with Crohn’s disease [1], 11% of patients with ulcerative colitis, and 5% of a control population. Elevated ASCA levels have been found in rheumatic diseases as well. In one study [2], 44.3% of Behcet’s patients with colitis were ASCA positive. Initial studies of ASCA levels in rheumatoid arthritis found that levels were elevated, but not to a statistically significant extent [3-4]. In another study from China [5], 40% of rheumatoid arthritis patients were positive for immunoglobulin A (IgA) ASCA and 20% positive for immunoglobulin G (IgG) ASCA, with control levels of 5.3% and 8.5%, respectively. The IgA ASCA levels were statistically significant.

The impetus for this case series came from a clinical observation by one of the authors several years ago - patients presenting with classic clinical and radiographic findings of rheumatoid arthritis, who were seronegative for rheumatoid factor and anti-cyclic citrullinated peptide (CCP) antibodies, could be positive for ASCA.

## Materials and methods

This study was conducted entirely at a single rheumatology center in Annapolis, Maryland without any outside funding. Between January 1, 2016 and January 1, 2018, all patients (n=241) with a diagnosis of rheumatoid arthritis were evaluated once for ASCA titer, rheumatoid factor, anti-CCP antibody, and antinuclear antibody (ANA) along with their routine laboratory studies. Specimens were sent to Quest Laboratories where the tests were performed using routine commercial assays. The ASCA assay was done using an enzyme-linked immunosorbent assay (ELISA) (Quanta Lite ASCA test (Inova Diagnostics, San Diego, CA, USA)) which can test for IgG and IgA antibodies to Saccharomyces cerevisiae (S. cerevisiae). This assay uses mannan, a beta-glucan or complex polysaccharide specifically found in the S. cerevisiae yeast cell wall. The patients were informed that the specimens were being drawn to address a specific research question and they provided their consent. All patients fulfilled the American College of Rheumatology (ACR) criteria for rheumatoid arthritis. No systematic attempt was made to test for celiac disease.

## Results

Two hundred and forty-one patients with rheumatoid arthritis from a single rheumatology center in Annapolis, Maryland were included in the study. They were divided into three groups:

1. ASCA +: Patients who were ASCA positive and could have either a positive rheumatoid factor, positive anti-CCP antibody, or positive ANA. At least one of these serologies was positive, but in many instances two of the serologies or all three serologies were positive.

2. ASCA -: Patients who were ASCA negative, but had a positive rheumatoid factor, positive anti-CCP antibody, or positive ANA.

3. Seronegative: Patients who were negative for ASCA, rheumatoid factor, anti-CCP antibody, and ANA. The ages of both ASCA positive and negative groups were similar as seen in Tables 1-2.

**Table 1:** The breakdown of the three study groups ASCA: anti-Saccharomyces cerevisiae antibodies.

Group	Number	Percent of total	Female	Male	Caucasian	Black	Other	Average age
ASCA +	158	65.6	107	51	127	28	3	62.3
ASCA -	74	30.7	56	18	55	15	4	64.8
Seronegative	9	3.7	4	5	8	1	0	64.2

**Table 2:** A summary of the serologies in the study population of rheumatoid arthritis patients ASCA: anti-Saccharomyces cerevisiae antibodies; RF: rheumatoid factor; CCP: cyclic citrullinated peptide; ANA: antinuclear antibody; SN: seronegative.

Group	ASCA	RF	Anti-CCP	ANA
ASCA + (n=158)	158 (100%)	78 (49.3%)	58 (36.7%)	47 (30%)
ASCA- (n=74)	0	70 (94.5%)	58 (78.3%)	23 (30%)
SN (N=9)	0	0	0	0

A higher percentage of ASCA negative patients were female (76%) as compared to the ASCA positive group (68%). In this study, 66% (158/241) of rheumatoid arthritis patients were positive for ASCA antibodies; 62% (148/241) were positive for rheumatoid factor, 48% (116/241) were positive for anti-CCP antibodies, 29% (70/241) were positive for ANA and 3.7% were negative for all serologies. Of note, 70 patients (29%) were positive for ASCA, but negative for rheumatoid factor and anti-CCP antibodies. Thus, 29% of these patients (70 of 241) would previously have been considered to have seronegative rheumatoid arthritis. This indicates that there is a distinct group of patients with clinical features of rheumatoid arthritis who are rheumatoid factor and anti-CCP negative, but who are ASCA positive. Notably, only 49% (78/158) of the ASCA positive patients with rheumatoid arthritis were positive for rheumatoid factor, whereas 95% (70/74) of the ASCA negative patients were positive for rheumatoid factor.

## Discussion

The expected percentage of rheumatoid arthritis patients positive for rheumatoid factor is 80%. Our figure of 62% in the total group of 241 rheumatoid arthritis patients is lower. We believe that this is representative of our referral patient population with rheumatoid arthritis. It should be noted that in the group of patients who were ASCA positive, the percentage of patients who were rheumatoid factor positive (49.3%) and CCP positive (36.7%) were reduced compared to the group of patients who were ASCA negative who had elevated rheumatoid factor (94.5%) and CCP (78.3%) positivity. This suggests there were serologic differences between the two groups. Also, one would expect about 20% of patients with rheumatoid arthritis to be seronegative, but when we excluded ASCA positive patients from the seronegative group, only 3.7% of the population was seronegative. Only three of the patients who were ASCA positive were known to have colitis.

Brewer's yeast is ubiquitous, being critical for the fermentation of beer, wine, and cheese; for baking bread and cakes; and for many other home and industrial processes. It is also present in several brands of probiotics. It occurs as a part of the gingival and gut microbiome. It is rare for S. cerevisiae to be a pathogen.

Our current paradigm is that autoimmune diseases, such as rheumatoid arthritis, are triggered by environmental factors in a genetically predisposed host. SKG mice spontaneously develop a T-cell mediated chronic autoimmune arthritis that is clinically and pathologically like rheumatoid arthritis. SKG mice fail to develop such an arthritis in a microbially clean environment, despite the presence of arthritogenic autoimmune T cells. Zymosan, a crude yeast cell wall extract, has been shown to trigger the development of severe arthritis in these SKG mice under sterile conditions [6]. In a remarkable series of experiments, Yoshitomi et al. found that beta-glucans from fungal cell walls could induce chronic arthritis in these mice. Blockade of dectin-1, a major beta-glucan receptor, prevented these mice from developing arthritis. Furthermore, when these mice, raised in an arthritis prone environment, were treated with antifungals, they did not develop arthritis [7] thus fulfilling Koch’s postulates. Further, beta-glucans, the main constituent of zymosan, have been shown to be the active moieties. Also, mannan from S. cerevisiae was able to induce arthritis in SKG Balb-c and SKG c57 black mice as well as evanescent arthritis in wild-type Balb-c mice.

In our study, we found that patients with rheumatoid arthritis have a high incidence of antibodies to mannan, a component of the S. cerevisiae cell wall. This suggests that in certain patients, as in the SKG mice, mannan might be important in inducing chronic arthritis.

## Conclusions

In this prospective study of 241 patients with rheumatoid arthritis, 66% were positive for antibodies to S. cerevisiae. The majority of patient with seronegative rheumatoid arthritis were found to be ASCA positive. In addition, 70 of 241 (29%) rheumatoid patients were negative for RF, CCP, and ANA but were found to be positive for either IGA or IGG ASCA. These results indicate ASCA may be a valuable test in assessing patients with rheumatoid arthritis, particularly if they were believed to be seronegative for rheumatoid factor and CCP. Hopefully, other investigators will find these results intriguing and pursue these findings in more scientifically rigorous studies.
